# A new psychotherapy that may treat PTSD in one session

**DOI:** 10.3389/fpsyt.2024.1440113

**Published:** 2024-11-04

**Authors:** Edmund Howe

**Affiliations:** School of Medicine, Uniformed Services University of the Health Sciences, Bethesda, MD, United States

**Keywords:** PTSD, brief, psychotherapy, new, anxiety, The Cortina Method, TCM

## Introduction

Patients who have post-traumatic stress disorder (PTSD) may not respond completely to standard treatments. As one text states, “Current therapeutic regimens are effective in a majority of patients, but many individuals continue to have persistent symptoms of PTSD despite conventional treatment” (1, 2). Thus, new approaches that can help these patients do better are needed. Accelerated resolution therapy (ART) and psychedelics with psychotherapy are relatively new therapies that are responsive to this need (3, 4). There is now also another psychotherapeutic approach that may be added to this list. It is based on the suggestion of New York University neuroscientists led by Daniela Schiller in *Nature* in 1910 that people’s fearful memories could be erased (5). This therapy is called The Cortina Method or TCM, named after Michael Cortina, who developed it.

This new approach to patients who have painful memories may be profoundly effective in some cases and is briefer than other interventions. As NBC has reported this and as is presented in this therapy’s website, just one visit is often enough to eliminate the crippling affect patients may have after trauma even when it has been present for decades. This relief may therefore wholly alter these patients’ lives. In light of this success, Cortina’s therapy has now been reported in over 400 press and media US outlets. Testimonials of both patients and therapists can be reviewed at MichaelCortina.com (accessed 27 May 2024). This opinion piece will present an overview of this therapy.

## Core merits of TCM

The core benefit from TCM likely involves memory reconsolidation. Memories previously stored may be rebuilt each time a memory is recalled, and as a result of this rebuilding, the affect associated with painful memories may be altered (6, 7). TCM, though, does not require patients to recall or recite their past trauma in detail. This benefit is potentially most important because, otherwise, patients may not initially seek out or may not later continue other PTSD therapies that could be beneficial, because they may find their reciting past details of their traumas too painful. As of now, this therapy has not shown any downsides, though in the future, follow-up studies may reveal these.

Patients responding have had the most painful emotions and tragic past traumas, yet after just one session, they have experienced relief. These traumas have included, for example, veterans who have had beloved service members blown up by an explosive device right next to them, patients who have had family members killed in a car accident while with them, and patients who have been gang-raped and even tortured. The gains from this therapy, it appears, too, may generalize. After experiencing this therapy, patients have reported relief of anxiety associated with other past traumas they have experienced. These findings have been recorded in video recordings that show these results from start to finish.

Peer-reviewed, double-blind research comparing TCM head to head with other, current standard therapies has not been done though. Long-term results have also not been obtained. Researchers interested in conducting these studies could, it would seem, readily conduct these studies. With the positive results obtained thus far and the absence of reported harmful side effects, though all this is only anecdotal, Institutional Review Boards likely would be prone to accepting pilot studies that are proposed.

Patients coming for treatment as shown in video recordings by therapists who have treated them often initially feel hopeless due to their anxiety having destroyed their quality of life for years when prior help has not succeeded. They have sometimes seen several past therapists without success. Since these dire beginnings and subsequent positive results are documented in numerous video recordings and reported by now large numbers of patients and therapists, in this piece, I will bring basic information regarding TCM to the attention of therapists and others.

## Examples of testimonials

Personal reports of patients and therapists are presently immediately available on the above TCM website. One patient exclaims there, for instance, “Unbelievable, incredibly life-changing for me! For 40 years I carried the past around as part of my identity, it’s not me anymore! Thank you!” Another says, “This session was more helpful than 20 years of therapy. It’s a miracle.” A therapist calls this therapy “truly life-changing.” Another therapist says that this method has “revolutionized how we deliver care for acute and complex trauma. The results are fast, lasting and transforming.”

Some of the therapists I talked to at the training session I attended were initially, themselves, patients. They had PTSD, learned of TCM, and then sought it out. After they regained their quality of life, some of these therapists, they told me, then, sought out TCM training to be able to offer it to their patients. Some therapists at this same training session I attended were returning for additional training. They sought to hone the skills they had learned only initially, since acquiring TCM skills is, as the case with most therapies, an asymptotic endeavor.

In TCM, Cortina has provided a panoply of interventions that he has integrated into a series of step-by-step measures that build upon each other. Counterintuitively, at least for me, therapists having attended even just one first training session may gain results close to those that Cortina himself achieved. The successes they may achieve, unprecedented in their practices, often surprise even them. This may occur because these interventions may be effective even when they are performed sub-optimally. Even when they use only some, these therapists report, these alone may still achieve for patients the results they have sought.

These interventions that Cortina both uses and teaches vary widely. They involve moving patients to use their visual imagination, enact bodily activities, change their cognitive frameworks, and engage in other activities unknown in other therapies. These last interventions may, for instance, require patients to do multiple tasks at the same time and respond in repetitive actions that have no conceptual meaning. All these interventions may have in common the potential and likelihood of enabling patients’ brains to become more open to change. The mechanisms of the gains from these procedures taken separately or together are not known and are, at best, speculative.

## The origin of TCM

How Cortina came to develop TCM may help explain this. He, as a social worker, had given patients state-of-the-art therapy but found this to be frustrating because, all too often, it was insufficiently effective. Consequently, he sought out from the medical literature all interventions that showed unequivocal evidence of therapeutic efficacy. He sought to maximize measures possible due to the plasticity of our brains. This therapy may also be effective because, in addition, it includes suggestions as used and taught early on by the psychiatrist Milton Erickson. He was known for his exceptional success in treating patients with whom other therapists had failed. Therapists continue to meet yearly to learn how to use and build upon his work until today (https://www.erickson-foundation.org/the-collected-works) (twelve volumes, accessed 19 March 2024).Erickson is seen and called by some as the father of indirect hypnosis. He would most often only talk with patients conversationally. His patients, like Cortina’s, come to feel safe and relaxed and, in this more suggestible state, are especially responsive to the suggestion that they can change. I studied with Erickson early on in my career and learned more about this means of helping patients from a colleague and psychologist, Harold Wain. He is an expert on hypnosis and at one time, for medical reasons, without using any anesthesia, talked a patient through surgery using only hypnosis. He has written this up and recorded this on film that I have seen (8). Cortina having also become aware of this intervention’s potential also then sought to use it.

Patients with PTSD have been shown to have higher levels of common cytokines. Evidence suggests that dysregulation of the mechanisms that regulate the HPA system results in lower levels of cortisol, and these lower levels can contribute to this chronic low-grade inflammatory state, and this inflammation may then drive symptoms of PTSD (9). It is unlikely that a single mechanism can explain how hypnosis may help some patients with PTSD overcome this. Hypnosis affects many parts of the brain differently, and its neural mechanisms are not at this time well understood (10).

## Discussion

I initially came to know of TCM through a colleague who contacted me, raving at how singularly successful she and others had found it to be. She, like me, had studied ART before this and found TCM to be even more rapid and effective. I went, then, myself, to a training session, as I had before to learn ART. I recall, to provide an example of ART for comparison, a patient who responded well to ART. She had repeatedly been sexually abused by an older person as a teen. She in her imagination using ART saw in her mind this abusing person decomposing into the dirt beneath her. This fertilized the soil, resulting in its growing beautiful flowers. She then saw herself giving these flowers to children who, upon receiving them, shrieked with delight.

TCM may equal or even surpass ART in its efficacy as my colleague believed, though, as I repeat, this has not been empirically studied and shown. TCM may most reflect and be seen as having built upon the views of Bessel Van Der Kolk as related in his book, *The Body Keeps the Score* (11). Passages in this work suggest the principles Cortina has adopted. I will include a few of these similarities here. Van Der Kolk reports, for example, how one patient feared that his approach was “some new age gimmick” (11, at 296). Some people, likewise, have thought this too when they have first heard from others examples of TCM’s most marked successes.

As a second example, Van Der Kolk relates the experience of a veteran who had unbearable nightmares. He saw in his dreams, for instance, Vietnamese children who had died. He reports in regard to this patient that trauma during war may not be treatable only with words. Cortina, in sync with this, uses visual, body, and other measures, also seeking to effect change by these additional means.

Van Der Kolk also cites the United States World Trade Center disaster as an example of how profoundly visual images may affect us. We may play sights of such disasters, he says, “over and over” in our minds (11, at 233). Seeing an event like this, he says, disrupts our brain’s networks of communications. Cortina, likewise, again, seeks to restore to patients the healthy brain circuits they had prior to their trauma.

Van Der Kolk finally summarizes the therapeutic ends he believes therapists should strive for. The sensory feelings of being seen, cradled, and supported, he says, can serve as antidotes to static, frozen feelings produced by prior trauma. They then can contradict these frozen feelings, replacing them with sensations rooted in “safety, mastery, delight and connection” (9, at 310). This could be seen as Cortina’s ultimate mantra just as well. His basic presupposition also mirrors Milton Erickson’s, that “people already have the resources they need to effect change” (12). Therapists, as others, may quite rightly question whether patients who have experienced life-shattering trauma can again ever escape life-deadening PTSD symptoms. They reasonably may doubt whether any therapy exists that can provide them relief. They may, after experiencing TCM, be without, however, their previously even unbearable anxiety.

TCM may restore meaningful life to patients who previously have given up hope and concluded that, for them at least, getting better will never be possible. One authority says that “New advances in the neurobiology of fear memory promise novel approaches to PTSD treatment, including the erasure of traumatic memories” (13). Still, of course, experts most appropriately urge caution. They say, for example, “Caution is required when inferring reconsolidation from behavioral results and when using reconsolidation theory to predict and account for such data” (14–17). Therapists seeking TCM training can pursue this through the above TCM website. Those wanting simply to become acquainted with this therapy may find that by just viewing this website, this will provide for them the information they want.

These patients’ and therapists’ testimonials particularly illustrate the profundity of the changes our brains’ plasticity may possibly make. For me, my merely becoming aware of even just these relatively few patients’ success has informed me of how much change is possible after only a brief therapy. Patients’ lives can be destroyed by trauma. Yet, they may have the potential, even then, to get better. I would not have, prior to coming to know of TCM, fully imagined this.

**Table 1** compares and contrasts TCM with exposure therapy, which is widely recognized as a standard of treatment for PTSD. This table was prepared by Michael Cortina.

**Table 1:** Comparison of exposure therapy and The Cortina Method (TCM).

Exposure Therapy	The Cortina Method (TCM)
Client is exposed to feared stimulus.	Exposure to feared stimulus not required.
Client relives pain.	Guest (client) does not have to relive any pain.
Requires 3 months of treatment with weekly sessions of 60–120 min; 8–15 sessions overall.	Requires one to two visits of 60–110 min.
Very anxiety-provoking for clients.	Guests report TCM being light, relaxing, even laughed and enjoyed the process.
Can be traumatic and make symptoms worse.	Worst-case scenario is it does not work. Nobody has gotten worse.
Can have high rate of dropout from treatment.	Zero to minimal dropout rate due to the efficiency of the treatment and the gentle, uplifting process of the treatment.
Effectiveness rate of 53%–80%.	Effectiveness rate of 90%.

## Author disclaimer

The opinions and assertions expressed herein are those of the author and do not reflect the official policy or position of the Uniformed Services University of the Health Sciences or the Department of Defense. Neither I nor my family members have a financial interest in any commercial product, service, or organization providing financial support for this research. References to non-federal entities or products do not constitute or imply a Department of Defense or Uniformed Services University of the Health Sciences endorsement. This work was prepared by a civilian employee of the US Government as part of the individual’s official duties and therefore is in the public domain and does not possess copyright protection (public domain information may be freely distributed and copied); however, as a courtesy, it is requested that the Uniformed Services University and the author be given an appropriate acknowledgment.
